# Artemisinin-based combinations versus amodiaquine plus sulphadoxine-pyrimethamine for the treatment of uncomplicated malaria in Faladje, Mali

**DOI:** 10.1186/1475-2875-8-5

**Published:** 2009-01-07

**Authors:** Kassoum Kayentao, Hamma Maiga, Robert D Newman, Meredith L McMorrow, Annett Hoppe, Oumar Yattara, Hamidou Traore, Younoussou Kone, Etienne A Guirou, Renion Saye, Boubacar Traore, Abdoulaye Djimde, Ogobara K Doumbo

**Affiliations:** 1 Malaria Research and Training Centre, Department of Epidemiology of Parasitologic Diseases, Faculty of Medicine, Pharmacy and Dentistry, University of Bamako, BP 1805, Bamako, Mali; 2 Malaria Branch, Division of Parasitic Diseases, National Center for Zoonotic, Vector-borne and Enteric Diseases, Centers for Disease Control and Prevention, Atlanta, GA, USA

## Abstract

**Background:**

Because of the emergence of chloroquine resistance in Mali, artemether-lumefantrine (AL) or artesunate-amodiaquine (AS+AQ) are recommended as first-line therapy for uncomplicated malaria, but have not been available in Mali until recently because of high costs.

**Methods:**

From July 2005 to January 2006, a randomized open-label trial of three oral antimalarial combinations, namely AS+AQ, artesunate plus sulphadoxine-pyrimethamine (AS+SP), and amodiaquine plus sulphadoxine-pyrimethamine (AQ+SP), was conducted in Faladje, Mali. Parasite genotyping by polymerase chain reaction (PCR) was used to distinguish new from recrudescent *Plasmodium falciparum *infections.

**Results:**

397 children 6 to 59 months of age with uncomplicated *Plasmodium falciparum *malaria were enrolled, and followed for 28 days to assess treatment efficacy. Baseline characteristics were similar in all three treatment groups. The uncorrected rates of adequate clinical and parasitologic response (ACPR) were 55.7%, 90.8%, and 97.7% in AS+AQ, AS+SP, and AQ+SP respectively (p < 0.001); after PCR correction ACPR rates were similar among treatment groups: 95.4%, 96.9%, and 99.2% respectively (p = 0.17). Mean haemoglobin concentration increased across all treatment groups from Day 0 (9.82 ± 1.68 g/dL) to Day 28 (10.78 ± 1.49 g/dL) (p < 0.001), with the greatest improvement occurring in children treated with AQ+SP. On Day 2, the prevalence of parasitaemia was significantly greater among children treated with AQ+SP (50.8%) than in children treated with AS+AQ (10.5%) or AS+SP (10.8%) (p < 0.001). No significant difference in gametocyte carriage was found between groups during the follow-up period.

**Conclusion:**

The combination of AQ+SP provides a potentially low cost alternative for treatment of uncomplicated *P. falciparum *infection in Mali and appears to have the added value of longer protective effect against new infection.

## Background

For more than five decades, chloroquine (CQ) has been the main drug for the treatment of uncomplicated malaria in Mali. The emergence of *Plasmodium falciparum *resistance to CQ has challenged control efforts in Mali, where recent studies indicate that the adequate clinical and parasitological response (ACPR) to CQ is estimated to be less than 50% in children aged between six months and nine years [1,2] in different endemic settings of the country. These data compelled the Ministry of Health to change to artemisinin-based combination therapy (ACT) for first-line treatment of uncomplicated malaria, in line with the recommendations of the Word Health Organization (WHO) [3]. The two combinations selected were artesunate-amodiaquine (AS+AQ) and artemether-lumefantrine (AL), which were found to be efficacious and well tolerated in the country [4,5].

Unfortunately, the implementation of ACT among the most vulnerable populations living in highly malarious rural areas has been hampered by the limited availability and high cost of the new drugs. In Mali, the healthcare system operates on local cost-recovery, and current prices for ACT in public sector range from 700–1,500 CFA (US$1.50–4.0). Thus, the majority of patients did not have access to the newly recommended treatment and continued to use chloroquine despite its low efficacy. Therefore, there was a need to test other combination therapies already available in the country before ACT became more widely available. WHO recommendations allow for the use of non-artemisinin combination treatment regimens for the treatment of uncomplicated malaria in settings where the component drugs are efficacious and well tolerated. Data from several settings have demonstrated a similar efficacy between ACT and the combination of amodiaquine plus sulphadoxine-pyrimethamine (AQ+SP) [6,7].

In Mali, SP is still efficacious (*in vivo *efficacy of ~95%) according to two separate studies [2,8] and is currently authorized only for intermittent preventive treatment during pregnancy (IPTp) [9]. There are data showing good efficacy (adequate clinical and parasitological response (ACPR) of 99.1%) of AQ in combination with AS [4]. We therefore hypothesized that the combination AQ+SP would be efficacious as suggested by earlier studies in other settings [10-12], and designed a three-arm *in vivo *efficacy study to evaluate this non-artemisinin containing combination and compare it with two artemisinin-containing combinations (AS+AQ and AS+SP).

## Methods

### Study site and population

The study was conducted in the rural village of Faladje, located 80 km Northwest of Bamako. The village has a Catholic mission health center that serves 23,000 inhabitants living in the village and surrounding areas. Malaria transmission is seasonal (July-October) with a peak in October. Most of the population is of the Bambara ethnic group with agriculture as the main occupation.

### Study design and procedures

This was a randomized single-blind trial, conducted between July 2005 and January 2006, that included children between six and 59 months of age living in Faladje. After written informed consent of patients' parents or guardians, study subjects were enrolled if they fulfilled the following WHO *in vivo *criteria [13]: i) microscope diagnosed mono-infection with *P. falciparum *with a parasitaemia of 2,000 – 200,000/μl, ii) axillary temperature of ≥ 37.5°C, iii) haemoglobin ≥ 5 g/dL, iv) absence of febrile illness caused by diseases other than malaria, vi) absence of danger signs (inability to stand or drink, convulsions, lethargy or persistent vomiting). Allocation to treatment groups was done according to block randomization (block size of 20); patients were not informed of the drug received.

Treatment was administered according to body weight at the following doses: AQ: 10 mg/kg/day from day 0 to day 2; AS: 4 mg/kg/day from day 0 to day 2; SP: 25 mg/kg of sulphadoxine and 1.25 mg/kg of pyrimethamine in single dose on day 0. All drugs were administered directly by the study team and the child was observed for 30 minutes. If vomiting occurred before 30 minutes, the dose was repeated; for vomiting after 30 minutes, a half-dose was administered. In the case of persistent vomiting, the child was referred to a health center for rescue treatment with intramuscular or intravenous quinine and withdrawn from the study.

After the day of enrollment (day 0), the child was assessed on days 1, 2, 7, 14, and 28. Parents/guardians were encouraged to come to the clinic at any time when the child was sick.

### Laboratory procedures

Capillary blood was obtained by fingerpick, thick and thin blood films were prepared and stained with 4% Giemsa for 20 minutes. Parasite densities were determined from thick blood smears by counting the number of asexual parasites per 300 WBCs assuming a WBC count of 7,500/μl. The same estimation method was used to calculate gametocyte density. Slides were read by an experienced medical microscopist, who was blinded to the treatment allocation. A negative blood film was defined as a slide with no parasites after review of 100 fields. Thin blood films were read only for parasite species determination. Ten percent of slides were read by a second reader, blinded to initial results. Discordant slides were read by a third microscopist.

Blood haemoglobin concentration was measured using a portable photometer (HemoCue: Anglholm, Sweden) on days 0, 14, 28 and any day of failure. Anaemia was defined as haemoglobin < 11.0 g/dl.

To distinguish recrudescence from new infection, molecular genotyping techniques were used for patients who failed after day 7. At enrollment and during the follow-up visits, blood spots were obtained on filter papers and evaluated at the Molecular Epidemiology and Drug Resistance Unit of the Malaria Research and Training Center in Bamako. Molecular analysis was done according to the following scheme: paired blots (from day 0 and the day of parasitaemia recurrence) were analysed by nested PCR of parasite merozoite surface proteins (MSP1 and MSP2) and microsatellite (CA1), to distinguish between new infection and recrudescence as previously described [5].

### Study endpoints classification

Treatment outcomes were classified following the 2003 WHO antimalarial drug efficacy guidelines [13]. Early Treatment Failure (ETF) was defined as one of the following: a) Danger signs/severe malaria on day 1, 2 or 3 with parasitaemia; b) parasite density on day 2 greater than at day 0; c) parasitaemia at day 3 with fever (axillary temperature ≥ 37.5°C); d) parasite density at day 3 equal or greater than 25% of that at day 0. Late Clinical Failure (LCF) was defined as danger signs/severe malaria or parasitaemia with fever occurring from day 4 to day 28 without previously meeting the criteria of ETF. Late Parasitological Failure (LPF) was defined as parasitaemia without fever from day 4 to day 28 and without previously meeting any criteria of ETF or LCF. An Adequate Clinical and Parasitological Response (ACPR) was defined as the absence of parasitaemia by day 28 without previously meeting the any criteria for ETF, LCF, or LPF. Outcomes measured were efficacy rates at day 28 with and without PCR adjustment. Other outcomes measured were anemia (haemoglobin < 11.0 g/dl) as well as fever, parasite, and gametocyte clearance times.

### Ethical approval

The protocol was approved by the ethical committee of the University of Bamako Faculty of Medicine, Pharmacy, and Odonto-stomatology (FMPOS) and the Institutional Review Board of the United States Centers for Disease Control and Prevention before the study was started. Overall community and local authorities' permission were also obtained in addition to parent or guardian informed consent, according to procedures described earlier [14].

### Data entry and analysis

Data were double entered using Microsoft ACCESS and statistical analysis was performed using SPSS version 11.0 (Chicago, IL, USA). Baseline characteristics of subjects among groups were compared using the chi-square test for categorical variables and Mann-Whitney *U*-test for non-normally distributed continuous variables. The primary analysis was per protocol. Proportion of patients failing treatment was compared across groups using the chi-square test. Paired t test was used to estimate haemoglobin increase from baseline to different time points of regular follow-up. To account for multiple comparisons the results of the tests were considered statistically significant when p < 0.017 (0.05 divided by 3).

## Results

**Figure 1:**
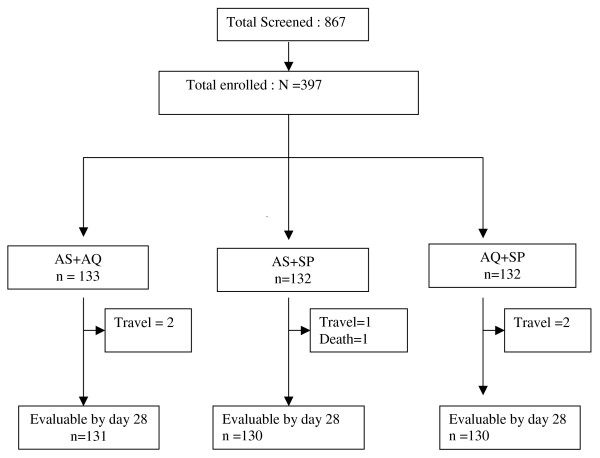
**Profile of children screened, enrolled, and completing study protocol**.

From July 2005 to January 2006, 876 children 6 to 59 months of age were screened for study entry (Figure 1); 397 were enrolled in the study and assigned to treatment arms (Table 1). Baseline characteristics were similar across the three treatment groups except for gametocyte carriage, which was more common among those in the AS+SP group. During follow-up, five children dropped out before day 14 because of travel, and one child whose parasitaemia had resolved died at day 7; the cause of death was unknown (Figure 1). There was no difference between groups regarding the rate of loss to follow-up. At day 28, 391 children were evaluable: 131, 130, and 130 in AS+AQ, AS+SP, and AQ+SP treatment arms respectively. Before PCR correction, ACPR was 55.7%, 90.8% and 97.7% in the AS+AQ, AS+SP, and AQ+SP arms respectively, and these differences were statistically significant (Table 2). After PCR adjustment, all treatment arms had an ACPR of > 95%, with no statistical difference among the groups (Table 2).

**Table 1:** Baseline characteristics of children enrolled in antimalarial *in vivo *efficacy study, Faladje, Mali.

Characteristics	AS+AQ N = 133	AS+SP N = 132	AQ+SP N = 132
Mean age ± S.D. (months)	34.49 ± 15.97	36.08 ± 15.97	37.56 ± 16.07
Range	6–59	6–59	8–59

Male gender, n (%)	72 (54.1)	64 (48.8)	82(62.1)

Mean weight ± SD (kg)	12.76 ± 3.30	12.77 ± 3.42	13.21 ± 3.49
Range	6.30–23.70	6–19.6	7.70–21.7

Mean height ± SD (cm)	92.05 ± 12.88	92.86 ± 14.18	94.39 ± 14.12
Range	63–115	64–120	65–133

Mean axillary temperature ± SD(°C)	38.59 ± .77	38.52 ± .78	38.58 ± .76
Range	37.5–40.2	37.5–40.6	37.5–40.8

Palpable spleen, n (%)	38(28.6)	40(30.3)	49 (37.1)

Mean haemoglobin ± SD (g/dl)	9.83 ± 1.80	9.71 ± 1.67	9.98 ± 1.59
Haemoglobin < 11 g/dl, n (%)	93(69.9)	102(77.3)	97(73.5)

GMPD/μl	24,113.4	22,846.8	24,051.7

Prior antimalarial use, n (%)	11(8.3)	4(3)	7(5.3)

Disease duration <= 1 day, n (%)	83(62.4)	92(69.7)	80(60.6)

Gametocyte carriage n (%)	1(0.8)	8 (6.1)	1(0.8)

AS+AQ, artesunate + amodiaquine; AS+SP, artesunate + sulphadoxine-pyrimethamine;

AQ+SP, amodiaquine + sulphadoxine-pyrimethamine. S.D, Standard deviation:

G/dl: Grams per deciliter

GMPD/μl: Geometric Mean Parasite Density per microliter.

P-values (not shown) were calculated from 2 × 3 *X*^2 ^test, (for categorical variables) and by KruskalWallis test (for continuous variables with non-normal distributions). Groups were not statistically different, except for gametocyte carriage (p = 0.006).

**Table 2:** Therapeutic efficacy of artesunate + amodiaquine, artesunate + sulphadoxine-pyrimethamine, and amodiaquine + sulphadoxine-pyrimethamine among children with uncomplicated malaria in Faladje, Mali

Characteristics	AS+AQ (N= 131)	AS+SP (N = 130)	AQ+SP (N = 130)
PCR uncorrected responses on day 28: n (%)			
			
Failures	58 (44.3)	12 (9.2)	3(2.3)
ETF	0 (0)	2(1.5)	0(0)
LCF	14 (10.7)	2(1.5)	1(0.8)
LPF	44 (33.6)	8(6.2)	2(1.5)
*ACPR	73 (55.7)	118 (90.8)	127(97.7)

PCR corrected responses on day 28: n; %			
			
Failures	6 (4.6)	4 (3.1)	1 (0.8)
ETF	0 (0)	2(1.6)	0(0)
LCF	2(1.5)	1(0.8)	0(0)
LPF	4(3.1)	1(0.8)	1 (0.8)
**ACPR	124(95.4)	125(96.9)	129(99.2)

AS+AQ, artesunate + amodiaquine; AS+SP, artesunate + sulphadoxine-pyrimethamine;

AQ+SP, amodiaquine + sulphadoxine-pyrimethamine

ETF, early treatment failure; LCF, late clinical failure; LPF, late parasitological failure; ACPR, adequate clinical and parasitological response.

Failures are the combination of ETF, LCF, and LPF. Percentages are rounded to achieve 100% of totals.

*X^2 ^test: p < 0.001, AS+AQ vs. AS+SP; p < 0.001, AS+AQ vs. AQ+SP; p = 0.016, AS+SP vs. AQ+SP

**X^2 ^test: p = 0.17 for the comparison of the three treatment arms.

At day 14, the proportion of children with anemia was significantly higher in AS+SP group (77.5%) when compared with AQ+SP (61.4%; p = 0.0046), but not with AS+AQ (65.9%; p = 0.030); the difference between the AS+AQ and AQ+SP groups was not statistically significant (Table 3). Overall, the mean haemoglobin was significantly increased in all treatment groups at day 14 (10.17 ± 1.47 g/dl) when compared with day 0 (9.84 ± 1.68 g/dl) (p < 0.01) and the greatest increase was observed among children treated with AQ+SP (0.45 g/dl) followed by AS+AQ (0.34 g/dl) and AS+SP (0.18 g/dl) (Table 3).

**Table 3:** Haemoglobin concentration, anaemia, and parasitaemia among participants in an antimalarial *in vivo *efficacy study, Faladje, Mali.

**Characteristics**	**AS+AQ**	**AS+SP**	**AQ+SP**
**Day 0**			
N	133	132	132
Mean Haemoglobin ± S.D (g/dl)	9.83 ± 1.8	9.71 ± 1.59	9.98 ± 1.59
Anaemia (< 11 g/dl), n (%)	93 (69.9)	102 (77.3)	97 (73.5)
**Day 14**			
N	131	130	130
^§^Mean Haemoglobin ± S.D (g/dl)	10.17 ± 1.5	9.89 ± 1.40	10.43 ± 1.49
*Anaemia (< 11 g/dl), n (%)	87 (65.9)	100 (77.5)	81 (61.4)
**Day 28**			
N	105	127	130
^§S^Mean Haemoglobin ± S.D (g/dl)	10.78 ± 1.49	10.5 ± 1.40	11.05 ± 1.52
**Anaemia (< 11 g/dl), n (%)	57(54.3)	76 (59.8)	60 (46.2)

**Parasitaemia, n (%)**			
^€^Day2	14(10.5)	14(10.7)	67(50.7)
^€^Day3	2(1.5)	1(0.8)	8 (6.0)
Day14	5(3.8)	2(1.6)	2(1.5)
^€^Day28	31(29.5)	9(7.1)	3(2.3)

AS+AQ, artesunate + amodiaquine; AS+SP, artesunate + sulphadoxine-pyrimethamine; AQ+SP, amodiaquine + sulphadoxine-pyrimethamine

*X ^2 ^test: p = 0.014 overall (2 × 3); p = 0.030, AS+SP vs. AS+AQ; p = 0.0046, AS+SP vs. AQ+SP; p = 0.48, AS+AQ vs. AQ+SP.

**X ^2 ^test: p = 0.08 overall (2 × 3); p = 0.027, AQ+SP vs. AS+SP; p = 0.21, AQ+SP vs. AS+AQ; p = 0.39, AS+AQ vs. AS+SP

^§^Paired t-test comparison day 0 vs. day 14: overall, p < 0.01; AQ+SP, p = 0.001; AS+AQ, p = 0.011; AS+SP, p = 0.033.

^§S^Paired t-test comparison day 0 vs. day 28: overall, p < 0.001; AQ+SP, p < 0.001; AS+AQ, p < 0.001; AS+SP, p < 0.001

^€^*X *^2 ^test: overall (2 × 3); Time points showing difference among the three groups: Day 2, p < 0.001; Day3, p = 0.017; Day 28, p < 0.001.

At day 28, the proportion of patients with anemia was 54.3%, 59.8%, and 46.2% in the AS+AQ, AS+SP, and AQ+SP groups respectively, but these differences were not statistically significant. Overall, the mean hemoglobin was significantly increased in all treatment groups at day 28 (10.78 ± 1.48 g/dl) when compared with day 0 (9.84 ± 1.68 g/dl) (p < 0.001); and the most significant increase was observed among children in AQ+SP (1.07 g/dl) group followed by AS+AQ (0.95 g/dl) and by AS+SP (0.79 g/dl) groups.

The proportion of patients with fever decreased from Day 0 (100%) to Day 1 (5.8%), Day 2 (2%), and Day 3 (1.5%) across all treatment arms. No difference was found among treatment groups at scheduled follow-up points with regard to the proportion of patients with fever. There were sharp declines in the proportion of children with parasitaemia from day 0 to day 2 in the AS+AQ (10.5%) and AS+SP (10.7%) groups, but not in AQ+SP (50.7%), (p < 0.001). By day 3, 1.5%, 0.8%, and 6% of patients were still parasitaemic in the AS+AQ, AS+SP, and AQ+SP groups respectively (p = 0.018) (Table 3).

**Figure 2:**
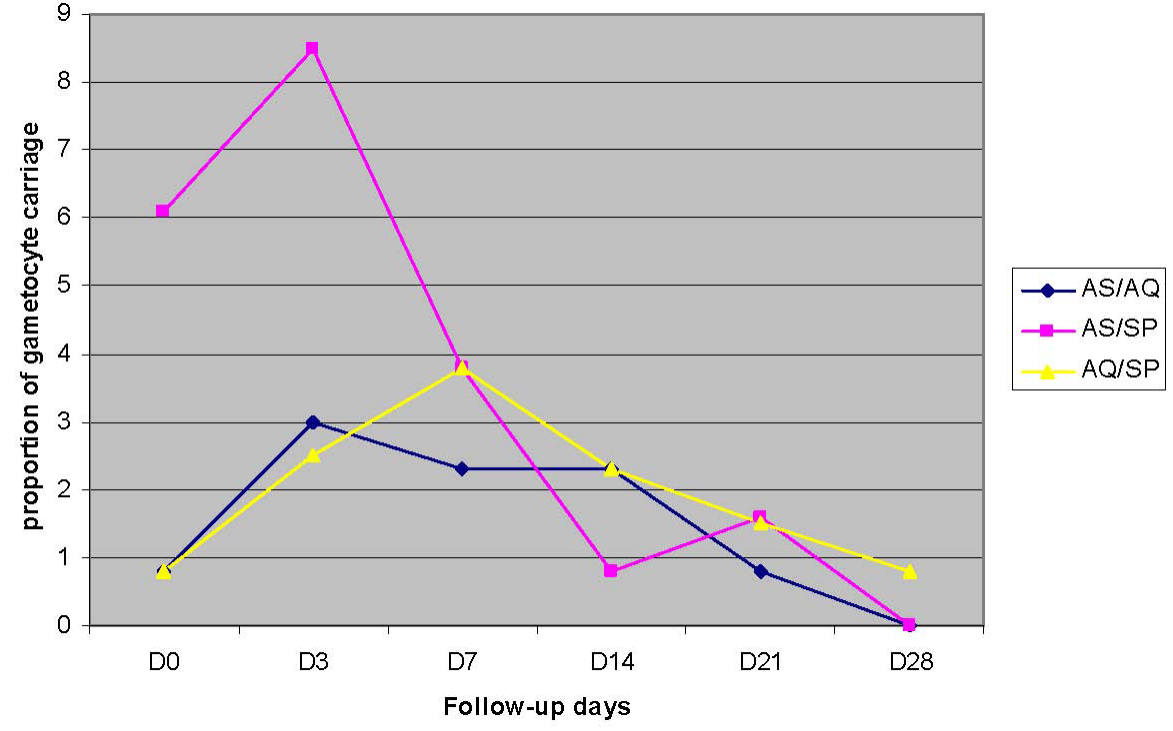
**Proportion of children with gametocytes from day 0 to day 28, in Faladje among children aged 6–59 months**.

From day 0 to day 3, there was an increase of the proportion of gametocyte carriage followed by a decrease from day 3 to day 28 where that proportion approached zero. No statistical difference in gametocyte carriage between study arms was found at day 3 (p = 0.03) or day 28 (p = 0.16) (Figure 2).

## Discussion

This study illustrated that the non-artemisinin containing combination AQ+SP was as efficacious as the artemisinin-based combinations AS+AQ and AS+SP for the treatment of uncomplicated *P. falciparum *malaria in children aged 6 to 59 months in Faladje, Mali. AQ+SP showed the highest ACPR at day 28 without PCR correction. After PCR correction, AQ+SP demonstrated the highest ACPR, although the difference was not statistically significant. These data corroborate those from other countries, which show the equal or superior efficacy of AQ+SP when compared with AS+AQ [15,16] or AS+SP [17]. These data suggest that AQ+SP provided greater protection against new infections during the 28-day follow-up period than either AS+AQ or AS+SP. The prevention of new infection provided by AQ+SP is probably due to the long elimination half-lives of the component drugs; while in AS+AQ, the arteminisin is quickly eliminated, providing no post-treatment prophylaxis [18].

Children treated with AQ+SP had a lower prevalence of anaemia on days14 and 28 than children treated with AS+AQ or AS+SP. Anaemia prevalence appeared to track with parasitaemia prevalence, suggesting that the combination of two longer half-life drugs in AQ+SP may provide longer protection against new infections and subsequent anemia. While mean haemoglobin concentration on day 28 was higher among children treated with AQ+SP, this difference was not statistically significant. This is in consistent with prior studies, which have demonstrated similar mean haemoglobin concentration between AS+AQ and AQ+SP [16,17].

The two artemisinin-based combinations, AS+AQ and AS+SP, showed faster parasite clearance than AQ+SP, but all three regimens resulted in prompt fever reduction. Despite the lower proportion of fever and parasitaemia observed during the first three days after treatment in the two ACT groups, there were more new infections in these groups than in the non-ACT group (AQ+SP). A greater risk of new infection following treatment with AS+AQ when compared with the AQ+SP has been reported earlier in low [12] and high transmission areas [11,19]. In Mali, AS+AQ, co-blistered but not co-formulated, has been introduced nationally as first-line therapy for uncomplicated malaria. However, there are concerns about patient compliance with the large number of tablets, and there is anecdotal evidence that patients may be taking the artesunate pills as monotherapy. With support from the Global Fund, the Malian Ministry of Health is providing AS+AQ free to children less than five years of age. Widespread implementation of ACTs in this vulnerable population should produce a significant reduction in malaria mortality and morbidity in Mali. However, it is essential to continue monitoring the efficacy of this regimen, and to consider alternatives should evidence of resistance develop.

## Conclusion

The combination of AQ+SP provides a potentially low-cost alternative for treatment of uncomplicated *P. falciparum *infection in Mali, and appears to have the added value of longer protective effect against new infections.

## Competing interests

The authors declare that they have no competing interests.

## Authors' contributions

In Mali, KK was the principal investigator and HM was the co-principal investigator of the study. At CDC, RN, MM, and AH contributed to the study design, protocol development, and manuscript writing. OY, HT, YK, EG, and RS participated in data collection, clinical investigation, and laboratory work. BT, AD were involved as field supervisors and laboratory data analysis. OD provided overall supervision in Mali. All authors participated in the writing and review of the final manuscript.
